# Chicago Gynæcological Society; Meeting July 16

**Published:** 1886-10

**Authors:** 

**Affiliations:** 2330 Indiana avenue


CHICAGO GYNÆCOLOGICAL SOCIETY.

*Regular Meeting, Friday, July 16, 1886.*—The VICE-PRESIDENT, HENRY T. BYFORD, M. D., in the chair.

I.—WAXHAM. *Discussion of paper on "Occlusion of the Os Uteri as an Impediment to Labour, with a Report of Two Cases."*

II.—BYFORD. *Discussion of paper entitled, "A Study of the Causes and Treatment of Pelvic Hæmatocele."*

I.—DISCUSSION OF PROFESSOR F. E. WAXHAM'S PAPER ON OCCLUSION OF THE OS UTERI AS AN IMPEDIMENT TO LABOUR; WITH A REPORT OF TWO CASES.

[Read at the May meeting.]

PROFESSOR W. W. JAGGARD said: From the very clear description of Dr. Waxham's case, I infer the condition was that described by Nägele under the term *conglutinatio orificii*, an uncommon complication of labour, but seldom indicating incision. Usually pressure of the finger is sufficient to open the os.

A more serious condition is that described by Schmitt, under the term *conglutinatio organica.* The cervical canal is obliterated to a variable extent. I had a case, illustrating this condition, under my observation in Professor Spaeth's wards during the winter of 1882. The lower half of the cervical canal was obliterated. Radial incisions were made around the *os externum*, and the canal was dilated with the index finger. Forceps were subsequently applied. The case was reported in the *Medical News*.

DR. JOHN BARTLETT said: I have nothing of interest to offer directly pertinent to the present discussion; but I have rather recently attended a case which I deem so nearly akin to those reported by Dr. Waxham as to justify me in mentioning it.

Mrs. Anderson, thirty-seven years old, came under my notice about three years ago. Five years since, she felt a burning pain in the nose and about the womb. At the same time her menstruation increased in quantity until in the course of a year it became profuse. Because of these difficulties, she sought relief from a quack. For the purpose of removing a cancer, which this pretender diagnosticated, a most violent caustic was put into the nostrils and applied to the womb. One year afterwards the tissues injured by the corrosive had healed; a violent uterine pain remained, and the flow had again become excessive. She applied to Dr. A. R. Jackson, who made an operation for the relief of the *atresia vaginæ* which he found existing. Although the operation was thorough, contraction recurred; so that when she was admitted into the Woman's Hospital in 1883, her condition was probably about the same as it was prior to Dr. Jackson's treatment. Dr. Mary H. Thompson operated upon the patient, opening thoroughly to the *os uteri.* Contractions, however, very soon reformed in the vagina; in October, 1885, her condition was serious. Her pulse was weak and frequent; neuralgic pains about the pelvis were nearly constant, and superadded to these older symptoms were those suggesting pregnancy. The uterus was enlarged and menstruation had ceased for three months. Of her condition at that time, Dr. Thompson writes: "The vagina was closed more perfectly than before. Not an opening could be seen in the occluding disc, which was only one inch from the *ostium vaginæ.* By examination through the rectum, it was ascertained that the uterus was enlarged, especially toward one side of the body." The distress of the patient, the apparently complete closure of the vagina, the non-appearance of the menses and the peculiar enlargement of the womb suggested either retained menstrual blood or some form of pregnancy. After a consultation, Dr. Thompson proceeded to open up the canal—at the time supposed to be perfectly occluded—between the uterus and vaginal *cul-de-sac.* In reality, a very small opening still existed; this was enlarged carefully by incision and distension until the *os uteri* was thought to be easily in communication with the remains of the vagina.

One month after the operation I was called upon to visit the patient. On the preceding night at a certain hour, most violent pains, as those of child-bearing, had come on, and had continued despite of anodynes, for some five hours. The pains had now, at the same hour as the night before, returned with increased violence. Not to go into details, I will say that the symptoms pointed strongly to some form of pregnancy. In view of the serious character of the case, I called in consultation on the next day, Dr. R. G. Bogue. We left the patient still in doubt as to her true condition. During the following night, I was again summoned; the exact resemblance of the pains to those of labor, and the now recognized hardening of the swelling above the pubes during these pains, made it quite certain that pregnancy existed, and that the contractions would finally lead to the extrusion of the fœtus from its sac *per vias naturales*, or otherwise. Upon careful examination, the vagina was found to be shut off about one inch from the *ostium* by a hard, firm and thick disc of cicatricial tissue. Toward one circumference the small opening detected and enlarged by Dr. Thompson a month before was recognized. By rectal examination, what seemed to be the *cervix uteri* was reached, three quarters of an inch beyond the upper face of the cicatricial disc. Connecting the disc and cervix was apparently a tube of tissue much smaller in circumference and thinner than proper vaginal walls. The pains continuing with regularity, Dr. Bogue and myself concluded to assist delivery. Slight incisions by means of the bistoury were made in the circumference of the opening in the cicatricial disc, a metallic dilator was then introduced, and when some dilatation had been effected, a modified Barnes's dilator of very small size was inserted. Within an hour, by the occasional use of the knife, and the continual tension of dilators, the disc opening admitted for a little distance the end of the finger into the remnant of the vaginal tube, above the disc. By the point of the finger pressed firmly onward, could now be recognized a hard body which was taken for the *cervix uteri.* With a little more dilatation of the disc opening, it was perceived that the hard body was the fœtus, and that the *os uteri* was healthy and dilating in a normal manner, the membranes being unruptured. As soon as the opposing disc opening was expanded to a size presumed to be sufficient to permit of the passage of the head, the membranes were ruptured. It was then discovered that the shoulder presented; by aid of suitable instruments the child was turned and in the somewhat too vigorous efforts at delivery the body parted from the head, the latter remaining *in utero.* This accident in such a case, with an extra, entirely rigid *os* precluding free procedure through the *os uteri*, ordinarily would be regarded as unfortunate. I looked upon it as a favorable step toward delivery, confident that by means of a suitable vectis the head could be easily scooped through both of the opposing *ora.* In fact, the head was readily so delivered, and the placenta falling over the *os uteri* was removed with the same instrument. One of the symptoms that confused the diagnosis on the first day of the appearance of labour pains was the unusually large, rapidly attained size of the suprapubic tumor. This symptom was now explained, for an examination to determine if the patient was entirely "cleared,"

revealed the presence of a second fœtus presenting by the head. The vectis was applied and the fœtus at once withdrawn, as was also in like manner the second placenta.

The labour revealed the true anatomy of the injured parts. The patient desired that the passage through the disc should be kept open, but inasmuch as the *os uteri* was almost immediately behind it, it was deemed useless and harmful to make the attempt. Within three months the dilated opening in the disc had contracted to a size but little greater than that observed before the miscarriage.

PROFESSOR F. E. WAXHAM said: I would simply allude to the great resemblance in the case coming under my care, between the uterine tissue and the fœtal membranes, especially in those cases in which there is but a small amount of amniotic fluid, and I can see how very easy it would be to do permanent injury to the mother by rupturing the uterine tissue by a pencil or some other sharp-pointed instrument, when, perhaps, by more extended and careful examination, it would be found that simple dilation would be sufficient. In the case reported, the knowledge that the amniotic fluid had been escaping for several hours was sufficient evidence to me that there was an *os*, and it was also proof that the tissues presenting were not membranes, but the uterine tissue. But the great trouble was to discover the *os*, and I assure you it was difficult indeed. The *os* was present in the center of the presenting mass, and yet we could not discover it. It was impossible for me to do so, and it was only after a continued, careful and searching examination that Dr. Nelson was enabled to detect the very slight dimple which was present.

II.—DISCUSSION OF DR. HENRY T. BYFORD'S PAPER, ENTITLED A STUDY OF THE CAUSE AND TREATMENT OF PELVIC HÆMATOCELES."

[Read at the June meeting.]

PROFESSOR T. D. FITCH said: I have had very limited experience with operative procedure in this class of cases. As a rule, I feel like praising the bridge that has carried me safely over. My usual treatment has been the expectant plan, or trusting to resorption of the clot. Resorption occurs in other tissues of the body, the leg or arm, where you would not think of opening the cavity and turning out the clot. It would be a very bad principle in surgery, I think. My experience has not been sufficient to condemn the operation entirely, but I feel like trusting to the safer plan of the expectant treatment. I have never operated in more than two or three cases, and would not have operated in them had not there been a mistake in diagnosis. One of these cases was a lady at Jefferson, who gave a history of cellulitis. There was softening and fluctuation in the tumor presenting. I was called in consultation by the attending physician. The symptoms were those of cellulitis, resulting in abscess. The aspirator was used and a very small amount of pus was drawn off, and then a larger amount of disintegrated blood. All was drawn off that could be, and the woman recovered, no bad results following the aspiration. No drainage was instituted, and no scooping out of the blood clot was performed; there was no special treatment except on general principles, and the vaginal injections of antiseptic fluids. The opening was not enlarged, the sac was not injected, nor washed out. The opening made by the aspirator needle probably closed up so that no air was admitted, and no decomposition or blood-poisoning occurred.

Another case was one in which I assisted in an operation for supposed extra-uterine pregnancy. Two distinguished Fellows of this Society were present and concurred in the diagnosis. It was decided to open the tumor through the vagina with the galvano-cautery knife, and when this was opened, there poured out of it a gelatinous fluid, as white, and as clear and pure as could be; it looked to me very much like soft boiled rice. It was a clear white, and perfectly inodorous. The sac was washed out with antiseptic fluids, and the patient treated on general principles; I think no drainage was used. The sac was not scooped out; nothing was turned out except the tablespoonful or two of gelatinous fluid of which I spoke.

Another case I might mention, in which the attending physician and myself (I was called in consultation) diagnosticated an abscess; opened it with the aspirator, and found that it was an hæmatocele. I believe the expectant plan of treatment is preferable to operative interference. I think a larger percentage of cases would recover under this treatment.

PROFESSOR JOHN BARTLETT said: I will take occasion to refer to a fatal accident that once came under my observation, which tends to show the necessity for the greatest care in opening cavities, *per vaginam*, whether resulting from hæmatocele or cellulitis. A patient was greatly reduced by long continued pelvic abscesses. It seemed to be one of those cases in which an operator is called upon to make a determined attempt to reach evacuate and curette a chain of abscesses found to exist within the pelvis. Several collections of matter were opened, and it was supposed that the object of the operative procedure had been happily accomplished. The final washing of the cavity with carbolized water was in progress, when suddenly the patient fell into a profound collapse; respiration ceasing and pulsation at the wrist failing. This condition was regarded as an accident from ether. Every effort at restoration was unavailing till a Faradic current was passed through the phrenic nerves at proper respiratory intervals. The patient then gradually rallied, and the danger was thought to have ceased. On the following morning the carbolized injection was repeated by an assistant; a fatal collapse immediately ensued. Post-mortem examination revealed a small opening through the roof of the pelvis, and the presence in the peritoneal cavity of the injected fluid. If the Society will pardon a digression, before closing I will take occasion to refer to a symptom of hæmatocele, which would seem to be as rare as it is suggestive. In one case, associated with this condition, I observed the whole surface of the abdomen below the navel, to present an ecchymotic appearance as from the extravasation of blood after an injury. The patient was alarmed at the "black and blue" appearance, regarding it as a sign of "mortification." It existed for weeks and disappeared, *pari passu*, with the pelvic extravasation.

PROFESSOR W. W. JAGGARD thought the ruptured cyst of extra-uterine pregnancy a more frequent cause of retro-uterine hæmatocele than the text-books would lead one to believe. Gallard has emphasized the importance of the operation of this etiological factor. He * makes a statement to the effect that independently of traumatism, almost all hæmatoceles are caused by the ruptured cyst of extra-uterine pregnancy. Such a broad statement naturally provoked salutary criticism. More recently, Veit †, of Berlin, has collected 146

* Leçons Cliniques des Maladies des Femmes. Paris, 1873. P. 635.

† Die Eileiterschwangerschaft. Stuttgart, 1884. P. 14.

cases of hæmatocele, of which forty cases, or 28 per centum, were probably due to the ruptured cyst of ectopic gestation. Veit's estimate does not appear extravagant.

He would like to inquire of the author of the paper, what was the indication in the case reported for operative interference? The indication had probably been stated, but through inattention he did not remember it. A small non-suppurating, retro-uterine hæmatocele of six months' standing was not, *per se*, an indication for any operative interference.

Any discussion of the surgical treatment of retro-uterine hæmatoceles, would be incomplete without some mention of Dr. A. Martin's plan of treatment in cases of extra-peritoneal hæmatoma. Laparotomy is performed, eventration of the intestines effected, the sac incised, evacuated and curetted, and subsequently united by sutures, drainage is maintained *per vaginam.* In Martin's hands, this operation has been perfectly successful in six cases.

PROFESSOR C. T. PARKES said: I do not think I have anything new to offer on the question of treatment of hæmatocele. My experience embraces only three cases. The first was a lady whom Dr. Fitch saw with me about a week after the initial symptoms, which present themselves in these troubles, had appeared, and we concluded to make an opening through the *cul-de-sac* of Douglas. I used the Paquelin cautery for the purpose of opening up the mass, which was not very extensive. The principal symptom which led us to think it was necessary to resort to interference, was the evidence of the presence of probable suppuration. The lady had been having slight chills and some corresponding rise of temperature, and we thought it best to be certain whether or no the mass had decomposed and broken down, so we opened it with the cautery, and quite a quantity of grumous, broken down blood, with clots came out. The lady was relieved of her pain and distress. We introduced a drainage tube, and through this tube passed a large catheter as long as the opening would permit, and washed out the cavity every day and followed it up for a long while, with a diminution in the size of the mass, until it got so that it was merely perceptible above the pubes, then the chills came on again more severely, and after suffering for a month or six weeks she finally died of septicæmia. In that case I was satisfied from the fact of being able to fill the cavity apparently, under the force of hydrostatic pressure, and then have something give way, and the fluid rapidly disappear, that we had a series of cavities which were opening into each other. I think if I had such a case to manage now I should do differently. I should use thorough antiseptic precautions, and care at present; such treatment was not then deemed necessary. The next case, a very interesting one, happened last winter; I saw the lady four or five weeks after she was taken ill. She was taken as though she were going to have a miscarriage after having missed menstruation twice, and when I saw her she was in an extreme condition of collapse; upon examining the abdomen, it was found full of something, dull on percussion, resonant above, and to the sides; on digital examination the ordinary signs of hæmatocele were present. This woman was in such a weak condition that I could not bring myself to the idea of interfering, and tried to support her and wait for events. I attended her two weeks, while she varied from one condition to another, all the time life hanging by a thread. In the third week, on examining her abdomen, I thought I detected fluctuation, and in two or three days was certain of it. I aspirated in the *linea alba* midway between umbilicus and pubes, and at first withdrew a quart of blood, but, although I was satisfied there was more there, I did not repeat the aspiration that day. Two days afterwards I aspirated again, and withdrew two quarts. She began to improve from that moment; I merely put her on tonics and supporting treatment; this was in February. I saw her about a month ago, and she was going about the house the same as anyone else. The third case was a little later in the same year, a lady who had been bleeding a little for some time, with the presence of signs of conception of two months' date. I made an examination, and was satisfied that I detected to the right of the uterus a mass as large as one's fist, easily reached by manipulation internally and externally, tense to the touch, and elastic. I diagnosed a probable hæmatocele, kept her quietly in bed, but did nothing special for her. The occurrence of this tumor was accompanied by extreme shock, prostration, pallor of the body and symptoms of collapse. She has now entirely recovered without any interference whatever. That last case led me to think of some of the reports I have read about surgeons being called to see a patient in collapse, finding she has flowed a little, with a history of probable pregnancy, making an examination, and discovering a little tumor, diagnosing extra-uterine pregnancy, using electricity and curing the patient. It seems to me there may be a possibility of there being a mistake in some of these cases of extra-uterine pregnancy that are cured so readily by the use of electricity. They are becoming very frequent. I must say, that it was a very difficult matter for me to decide in this case, whether it was extra-uterine fœtation or hæmatocele; still I am satisfied that it was an hæmatocele.

DR. H. T. BYFORD said: Before closing the discussion, I would like to add the following case to the series reported in the paper.

CASE VI.—Mary H., a German servant girl, 25 years old, was taken sick with pains about the lower abdomen, nine months ago. The attack, which came on after a menstrual period, kept her in bed little of the time, but did not pass off. In six weeks, her menses came on and lasted two weeks. The bleeding ceased for a few days, then returned and had continued, in varying quantity, until stopped by ergot about a week before I saw her. Vesical irritation was an almost constant symptom. Up to that time she had tried to attend to her work, but then gave up her place. She told me, a little over a month ago, when I first saw her, that she had felt worse since taking the medicine. The great pelvic tenderness subsided rapidly under the "absolute rest" treatment, and in less than a week afterwards, I was able, without paining her, to completely circumdigitate a large boggy or semi-elastic tumor in the right broad ligament, extending behind the uterus from a level with the internal *os* upwards, and reaching into the left broad ligament, where it felt harder and nodulated. The uterus was antiflexed, displaced anteriorly, and to the left, (leaving only room enough between the cervix and the pubes for the index finger), and intimately attached to the surrounding mass. The probe entered three inches, turning forwards. After keeping off her feet, although not in bed, using hot douches, iodine applications to the abdomen, iron internally, and having glycerine plugs applied about every three days for three weeks, the tumor had become harder, somewhat nodulated in places, and perceptibly smaller. She had felt quite well again until the last few days, when she undertook to resume her domestic duties.

This case shows well the positive benefit of rest, and the positive harm that is sure to result from want of it. Its history is similar to the history of many such tumors which go on to suppuration, but which, with proper treatment, would have been promptly absorbed.

The unfortunate case related by Dr. Bartlett bears witness to the dangers of the curette in pelvic hæmatoceles, and is probably one among many somewhat similar ones that have not been reported. The necessity of a large opening, perfect drainage and great antiseptic precaution is vividly shown by one of the cases recited by Dr. Parkes. His view as to the liability to the formation of pus pockets is corroborated by the sudden discharge of half an ounce or more of pus on March 26, in the case of Mary H——, followed by the rapid sinking of the uterus back into a natural position. This pus pocket, had the operation *not* been performed, would probably have formed and pointed upwards in the direction of the least resistance, and would have become an abdominal abscess, and a serious thing to manage. I quite agree with Dr. Parkes that simple hæmatoma and hæmatocele are too often thought to result from extra-uterine pregnancy, and think it is partly the result of Gallard's theory that all non-traumatic cases are extra-uterine pregnancies, a theory which has done its good and has had its day. The intensity and persistance of the local symptoms, the passage of the decidua, and the past or present characteristic symptoms of the pregnant condition should usually prevent such a mistake.

I think, with Dr. Jaggard, that Bandl would have us operate too early; I only claimed that Bandl's views were a great advance in the therapeutics of pelvic effusions, in that, while recognizing the dangers of early interference, he does not allow the fear of inducing septicæmia to intimidate him into waiting until septicæmia has already accomplished its mischievous and perhaps fatal work. The reason why Bandl's latest views have had so little apparent effect upon the profession, is that they have only been before the profession at large for a few months. I had come to the conclusion that with our present knowledge of antiseptics we need not be frightened out of opening up these accumulations, and had acted upon it before I knew of Bandl's views; and so had many others whose veneration for long established authority had not overpowered their individual judgment.

A. Martin's method of operating for hæmatoceles and hæmatoma is *one* method, but that it is *the* method cannot be maintained upon scientific grounds so as to convince the profession; nor has it as yet been so proved by its success. As to the frequent bunglesomeness of operations *per vaginam* and *per rectum*, there is scarcely to be found an opportunity for the bungler like the performance of laparotomy for pelvic disease. I doubt if I exaggerate in saying that half of the abdominal sections are done in a bungling manner, especially when compared to those of Martin and a few others.

In my paper I advocate the expectant plan of treatment, and have used it, and so far succeeded with it, in all of this series of cases except one. That case was operated upon because the conditions for a cure without an operation were not attainable; because even if attainable, they would have taken too much time to restore the patient to usefulness; and because if properly done, the operation in such a case is almost devoid of danger. I regard it as a good illustration of when we may operate in case the expectant plan does not afford relief. In Case VI, Mary H., which I have just reported, I shall use every effort to do without surgical interference, because the interior of the sac cannot be easily and safely reached.

Protheroe Smith, M.D., M.R.C.P., of London, was then elected Honorary Fellow of the Society.

W. W. JAGGARD, M.D., *Editor*.

2330 Indiana avenue, September, 1886.

